# Association of COVID-19 preventive behavior and job-related stress with the sleep quality of healthcare workers one year into the COVID-19 outbreak: a Japanese cross-sectional survey

**DOI:** 10.1186/s13030-024-00304-w

**Published:** 2024-03-06

**Authors:** Muneto Izuhara, Kentaro Matsui, Ryo Okubo, Takuya Yoshiike, Kentaro Nagao, Aoi Kawamura, Ayumi Tsuru, Tomohiro Utsumi, Megumi Hazumi, Yohei Sasaki, Kazuyoshi Takeda, Hirofumi Komaki, Hideki Oi, Yoshiharu Kim, Kenichi Kuriyama, Takeshi Miyama, Kazuyuki Nakagome

**Affiliations:** 1 Department of Sleep-Wake Disorders, National Institute of Mental Health, National Center of Neurology and Psychiatry, Kodaira, Tokyo Japan; 2 Department of Clinical Laboratory, National Center Hospital, National Center of Neurology and Psychiatry, Kodaira, Tokyo Japan; 3 Clinical Research & Education Promotion Division, National Center of Neurology and Psychiatry, Kodaira, Tokyo Japan; 4 Department of Psychiatry, National Center Hospital, National Center of Neurology and Psychiatry, Kodaira, Tokyo Japan; 5 Department of Public Mental Health, National Institute of Mental Health, National Center of Neurology and Psychiatry, Kodaira, Tokyo Japan; 6 Department of Behavioral Medicine, National Institute of Mental Health, National Center of Neurology and Psychiatry, Kodaira, Tokyo Japan; 7 Department of Surgery, National Center Hospital, National Center of Neurology and Psychiatry, Kodaira, Tokyo Japan

**Keywords:** Work environment stress, Financial instability, Working remotely, Sleep quality, Poor sleep

## Abstract

**Background:**

This study aimed to evaluate the association of COVID-19 preventive behavior and job-related stress with sleep quality among healthcare workers (HCWs). We conducted a cross-sectional survey using a questionnaire at the National Center of Neurology and Psychiatry, Tokyo, Japan.

**Methods:**

A total of 586 participants who completed the questionnaire were eligible for the study. The Pittsburgh Sleep Quality Index was used to evaluate sleep quality. We examined the level of engagement between poor sleep and COVID-19-related infection preventive behaviors, such as avoiding closed spaces, crowded places, and close contact (three Cs), a distance of at least one meter from others, wearing a face mask regularly, washing hands regularly, and working remotely, as well as job-related stress in the work environment, exposure to patients, potential risk of infection, fear of infecting others, need for social confinement, and financial instability. We conducted a hierarchical logistic regression analysis to examine the relationship between poor sleep and COVID-19 preventive behavior, job-related stress, and other covariates, including age, sex, and the Kessler Psychological Distress Scale (K6), which was used to measure non-specific psychological distress.

**Results:**

Poor sleep was observed in 223 (38.1%) participants. Adherence to COVID-19 preventive measures was relatively high: 84.1% of participants answered “always” for wearing a face mask regularly and 83.4% for washing hands regularly. In the multivariate logistic regression analysis, stress in the work environment (odds ratio [OR] = 2.09, 95% confidence interval [CI], 1.37–3.20; *p* < 0.001), financial instability (OR = 1.73, 95% CI, 1.12–2.67; *p* < 0.05), and low adherence to working remotely (OR = 1.65, 95% CI, 1.06–2.57; *p* < 0.05) were independently and significantly associated with poor sleep after controlling for the covariates.

**Conclusions:**

One year into the COVID-19 pandemic, the poor sleep rates of HCWs remained high. These results emphasize the need to protect HCWs from work environment stress and financial concerns.

**Supplementary Information:**

The online version contains supplementary material available at 10.1186/s13030-024-00304-w.

## Background

Sleep was reported to be essential for maintaining mental and physical health during the COVID-19 pandemic [1]. Sleep disruption exacerbates mental health problems, such as increased stress responsivity and emotional distress [2]. Various studies have consistently reported the increased prevalence of disrupted sleep, especially among healthcare workers (HCWs) during the pandemic [3, 4]. Physicians and nurses had a high rate of insomnia of approximately 31% (95% confidence interval [CI]: 27–36%), compared with that of the general population (18%, 95% CI: 15–21%).^5^ During the COVID-19 pandemic, HCWs have undergone work-related stress, such as coming in contact with patients with COVID-19.

Before the development of COVID-19 vaccines (first approved on 14th December 2020 by the US Food and Drug Administration), approximately 5% of patients with COVID-19 and 20% of those hospitalized experienced severe symptoms necessitating intensive care. Over 40% of deaths were reported among patients in intensive care units [5]. Our previous study reported that approximately 80% of HCWs reported increased psychological burdens and that 50% reported physical burdens [6]. However, the factors contributing to poor sleep during the COVID-19 pandemic have as yet not been established.

Job-related COVID-19-related stresses were unavoidable for HCWs: fears of infection, death, spreading of infection to others, contact with potentially infected patients, and social confinement due to close contact with patients with COVID-19. The financial challenges faced during the pandemic may have also affected the sleep quality of HCWs. Another possible factor contributing to poor sleep quality is the preventive behaviors taken against COVID-19. Implementing COVID-19 precautions in the workplace was associated with increased fear and worry [7], while personal protective equipment became a physical burden [8]. In contrast, washing hands, one of the most common precautionary practices, has been reported to be associated with fewer symptoms of sleep disturbances [9]. To protect HCWs from sleep disturbances in the case of pandemics in the future, it would be essential to identify the factors associated with sleep disturbances. Therefore, we conducted a cross-sectional survey of HCWs to examine the association between poor sleep, adherence to preventive measures, and job-related stress during the COVID-19 pandemic.

## Methods

### Study design

We conducted a cross-sectional survey on HCWs between February 24–26, 2021, at the National Center Hospital, National Center of Neurology and Psychiatry (NCNP), Tokyo, Japan. The NCNP is one of the National Research and Development Agencies of Japan established on the concept that the hospital and research institutes work together to conduct research and development aimed at overcoming mental, neurological, muscle diseases, and developmental disorders, to provide advanced pioneering medical care based on the research results, and to disseminate good care throughout the country. The NCNP hospital consists of an 11-unit ward with a total of 486 beds. During the pandemic, one of these units, with 41 beds, was designated and used as a special unit for psychiatric patients with concurrent COVID-19 infection.

### Assessment of sleep quality and disturbances

The participants’ sleep was evaluated using the Japanese version of the Pittsburgh Sleep Quality Index (PSQI) [10, 11]. The PSQI measures sleep quality and disturbance over the prior 4-week period based on a self-rated evaluation [10]. The 19 PSQI questions evaluate seven categories of sleep: quality, latency, duration, efficacy, disturbances, hypnotic use, and daytime dysfunction. Domain scores are coded from 0 to 3; a global PSQI score was obtained by summing the domain scores. The global PSQI score ranges from 0 to 21, and a score of > 5 reflects poor sleep. The sensitivity and specificity of the Japanese version of the PSQI are 85.7% and 86.6%, respectively, for primary insomnia [11]. In this study, we defined a poor sleeper as a participant with a PSQI global score greater than 5.

### Measurements of covariates

We also examined job-related stressors during the COVID-19 pandemic due to (i) the work environment in terms of facility policy, collaboration with colleagues, and non-medical work; (ii) exposure to patients; (iii) potential risk of COVID-19 infection; (iv) fear of infecting others; (v) social confinement; and (vi) financial instability, which participants rated on a 5-point scale as “never,” “slight,” “moderate,” “severe,” and “very severe.” We examined adherence to COVID-19 infection preventive behaviors, according to previous studies [12, 13], including (a) avoiding closed spaces, crowded places, and close contact (three Cs) [14]; (b) maintaining a distance of at least one meter from others; (c) wearing a face mask regularly; (d) washing hands regularly; and (e) working remotely. Participants rated items as 1, 2, 3, or 4 for “always,” “usually,” “occasionally,” and “never,” respectively.

We evaluated previously detected poor sleep-related factors during the COVID-19 pandemic [15, 16], including sex, age, body mass index (BMI), smoking habits (using a 6-point scale: never, quite, occasionally, less than 10 cigarettes per day, 11–20, and 21 or more), alcohol consumption (for frequency, using a 6-point scale: never, less than once per month, one to three times per month, one or two times per week, three or four times per week, and five or more times per week; for amount, using a 6-point scale: less than 10 g of ethanol equivalent, 10–19 g, 20–39 g, 40–59 g, 60–79 g, and 80 g or more), exercise habits (using a 7-point scale: never, less than 30 min, 30–59 min, 60–119 min, 120–179 min, 180–239 min, and 240 min or more than 240 min [per week]), the number of people living with the participant, burden of caring for the elderly or children, type of profession (doctors, including physicians and dentists; nurses; other medically qualified professionals; and non-medically qualified professionals), average working hours per day (using a 8-point scale: 6 h or less, 7, 8, 9,10, 11, 12, and 13 h or more), being a frontline worker (experience of high risk of exposure to airborne droplets including intubation, specimen collection from patient’s nose or throat, and surgical operation), history of close contact with patients with COVID-19 (using a 3-point scale: yes, no, or not sure), experience of self-confinement due to possible infection of COVID-19 (yes or no), frequency of using public transportation (using a 4-point scale: less than one time per week, one or two times, three or four times, and five times or more), experience of discrimination due to being an HCW (yes or no) [6], and existence of chronic disease (yes or no; if yes, specify: hypertension, diabetes mellitus, lung disease including chronic obstructive pulmonary disease and asthma, heart failure, stroke, cancer, and others). As an association between non-specific psychological distress and poor sleep has been previously reported [17], we evaluated non-specific psychological stress by using the Kessler Psychological Distress Scale (K6) [18]. The K6 (6 domains, range 0–24) assesses nervousness, hopelessness, restlessness, depression, effort, and worthlessness over 30 days. Scores of 13 or higher indicate serious psychological distress [19]. The sensitivity and specificity of the Japanese version of the K6 with a cut-off score of 12/13 are 64.7 and 97.3%, respectively, for non-specific distress [20]. In this study, we defined non-specific psychological stress as participants with a 13 or higher score on K6.

For simplicity, we divided the socio-demographic variables into two groups. Participants with smoking habits were categorized as non-regular smokers (including never, quit, and occasionally) and regular smokers (including fewer than 10 cigarettes, 11–20 cigarettes, and 23 or more cigarettes daily). Participants with alcohol consumption were categorized as habitual drinkers (40 g or more alcohol consumption per day) and occasional/non-drinkers (40 g or less alcohol consumption per day), according to a previous study that suggested that alcohol consumption of less than 40 g per day might not affect sleep [21, 22]. We dichotomized the participants depending on their exercise habits: regular exercisers (120 min or more a week) and non-regular exercisers (119 min or less a week), according to a previous study, which showed that exercising for 150 min or more per week decreases mortality [23]. Further, working status was also dichotomized: participants engaged in long working hours (9 h or more) or normal working hours (8 h or less), according to a previous study, which showed that working for 9 h or more deteriorates sleep quality [24]. For infection control measures, we used the median value and divided the groups into two [25], owing to the lopsided distributions of the variables. For job-related stress, the participants who reported “severe” or “very severe” stress were assumed to be highly stressed out [26]. 

### Statistics

We conducted a hierarchical logistic regression analysis to explore the factors contributing to poor sleep. First, all variables were examined initially with univariate models (crude model). Second, we conducted a hierarchical logistic analysis for all variables that were significantly correlated in previous studies to determine the main correlates controlling for confounding factors, including age, sex, non-specific psychological distress (as measured by the K6), existence of chronic diseases, being a frontline worker, and burden of caring for the elderly or children [15, 16]. We conducted a three-layer hierarchical logistic regression analysis. Adjusted 1: including age and sex. Adjusted 2: variables in Adjusted 1 + previously detected poor sleep-related covariates, including the existence of chronic diseases, being a frontline worker, and the burden of caring for older adults or children, other than non-specific psychological distress, because stress has long been considered to be one of the major factors contributing to poor sleep [27]. Adjusted 3: variables in Adjusted 2 + non-specific psychological distress.

We conducted a complete case analysis excluding individuals with missing values. We created a crosstabulation table using the variables associated with poor sleep in the univariate model logistic analysis to evaluate the relationship between types of profession and poor-sleep related factors. Previous studies indicated that most effect sizes of factors associated with COVID-19 preventive behaviors were small to medium [28]. With a small effect size (d = 0.2), a power analysis indicated that a sample size of *N* ≥ 541 was required, with 95% power and an alpha level of 0.05. SPSS v23 (IBM Corp, Armonk, NY, USA) was used for statistical analysis. The level of significance was set as *p* < 0.05.

## Results

**Fig. 1:**
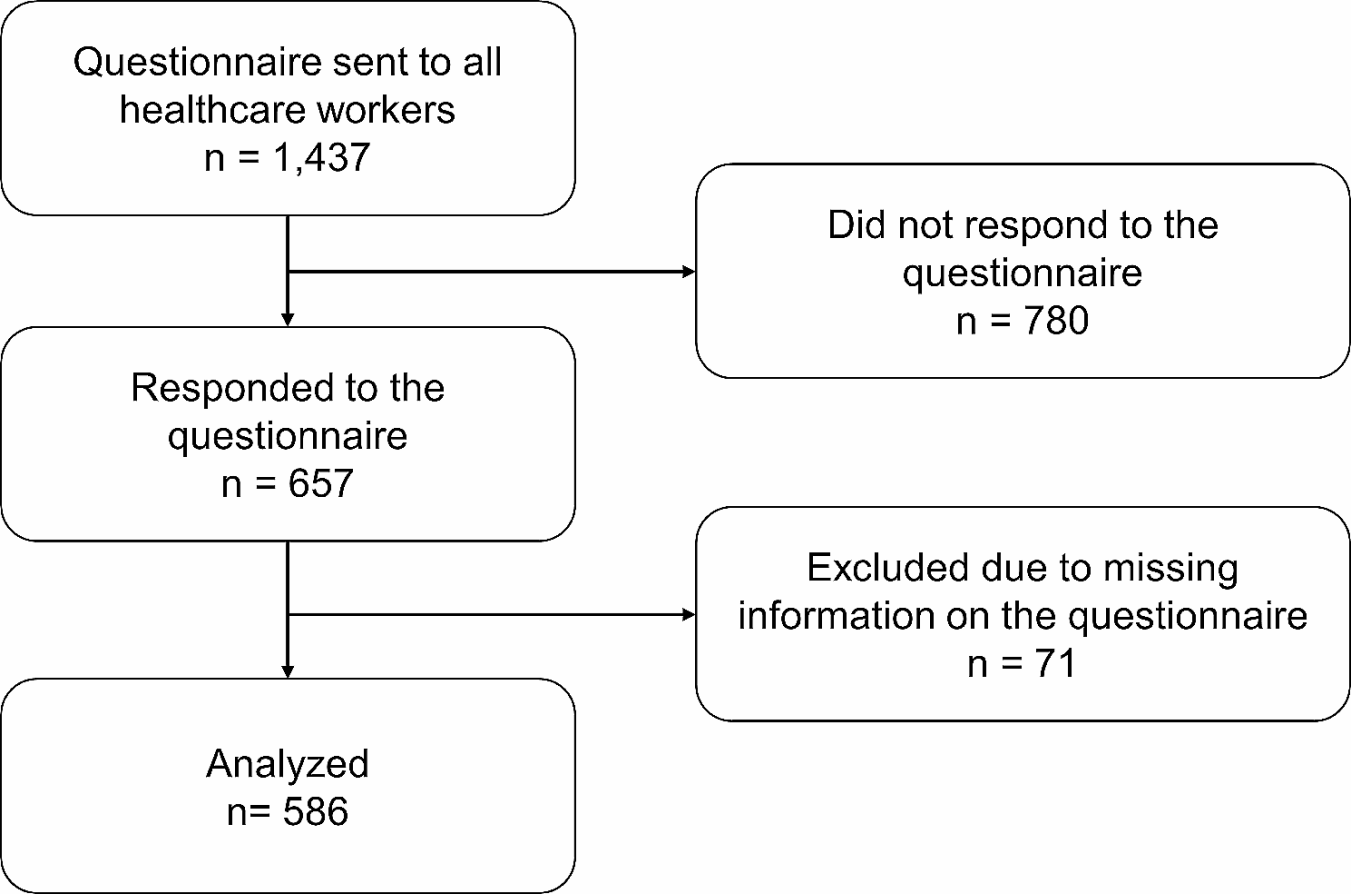
Study flowchart

A total of 1,437 HCWs at the National Center Hospital, NCNP, Tokyo, Japan, were invited to participate in this study, and 657 (45.7%) agreed. After excluding 71 who did not complete the questionnaire, 586 were included in this study, comprising 42 (7.2%) physicians or dentists, 172 (29.4%) nurses, 116 (19.8%) other medically qualified professionals, and 256 (43.7%) non-medically qualified professionals (Fig. 1). Poor sleep (6 or higher PSQI global score) was noted in 223 (38.1%) participants, 363 (61.9%) showed a 5 or lower PSQI global score (without poor sleep), and most were female (*N* = 396, 67.6%).

**Fig. 2:**
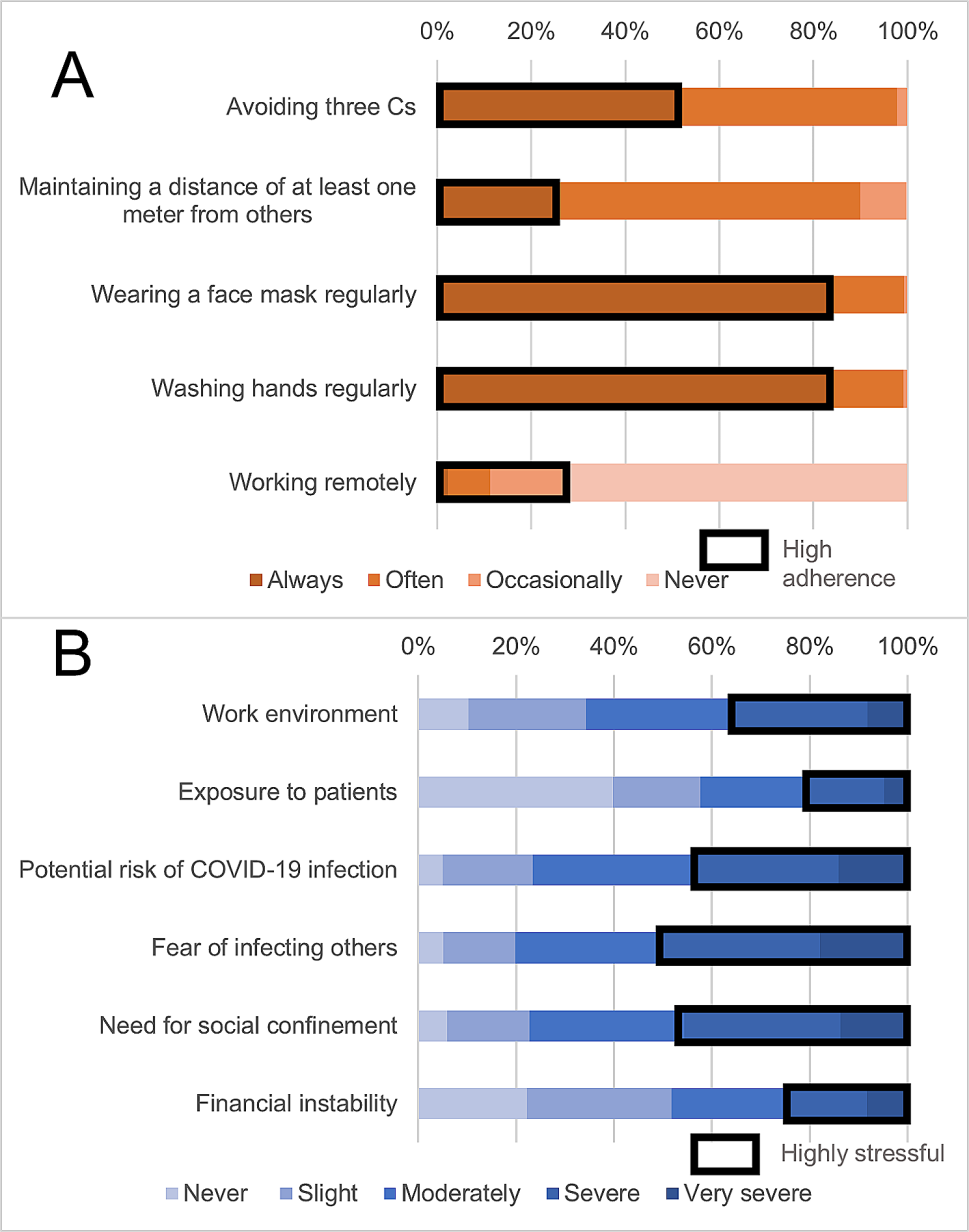
COVID-19 preventive behaviors and job-related stress during the COVID-19 pandemic. **A**: COVID-19 preventive behavior: Participants who answered “always” were considered to show high adherence to protocols for avoiding the three Cs (51.4%), maintaining a distance of at least one meter from others (25.6%), wearing a face mask regularly (84.1%), and washing hands regularly (83.4%), while participants who answered “always,” “often,” or “occasionally” were considered to have high adherence to work remotely (28.0%). **B**: Job-related stress under the COVID-19 pandemic: Participants who answered “severe” and “very severe” were considered to be highly stressed: in the work environment (35.7%), from exposure to patients (21.0%), from the potential risk of COVID-19 infection (43.9%), from the fear of infecting others (50.5%), by the need for social confinement (45.6%), and from financial instability (25.1%). *Abbreviations* Three Cs, closed spaces, crowded places, and close contact

The median (interquartile range) age of the participants was 43.0 years (34.0–50.0). Median (interquartile range) BMI was 21.5 (19.9–23.7). Only 39 (6.7%) participants were regular smokers, whereas 190 (32.4%) were habitual drinkers. A total of 169 (28.8%) participants had chronic diseases (Additional File 1). The following participants were considered to show high adherence to each of the COVID-19 preventive behaviors: those who answered “always” for avoiding the three Cs (51.4%), maintained a distance of at least one meter from others (25.6%), wore a face mask regularly (84.1%), washed their hands regularly (83.4%), and “always,” “often,” or “occasionally” worked remotely (28.0%) (Fig. 2). For COVID-19-related stress, participants who reported “severe” or “very severe” stress were considered to be highly stressed out: stress in the work environment (35.7%), exposure to patients (21.0%), with a potential risk of COVID-19 infection (43.9%), fear of infecting others (50.5%), need for social confinement (45.6%), and financial instability (25.1%).

A univariate logistic regression analysis showed that 14 items were significantly associated with poor sleep: older age (odds ratio [OR] = 1.02; 95% confidence interval [CI], 1.01–1.04; *p* < 0.01), higher BMI (OR = 1.06, 95% CI, 1.01–1.11; *p* < 0.05), being a frontline worker (OR = 1.73, 95% CI, 1.03–2.89; *p* < 0.05), existence of chronic diseases (OR = 1.66, 95% CI, 1.16–2.39; *p* < 0.01), experience of discrimination due to being an HCW (OR = 2.10, 95% CI, 1.08–4.07; *p* < 0.05), higher adherence to protocols for avoiding the three Cs (OR = 0.70, 95% CI, 0.50–0.97; *p* < 0.05), lesser opportunity of working remotely (OR = 1.72, 95% CI, 1.17–2.53; *p* < 0.01), stress in the work environment (OR = 2.86, 95% CI, 2.01–4.06; *p* < 0.001), exposure to patients (OR = 1.83, 95% CI, 1.22–2.73; *p* < 0.01), potential risk of COVID-19 infection (OR = 1.98, 95% CI, 1.41–2.78; *p* < 0.001), fear of infecting others (OR = 1.81, 95% CI, 1.29–2.54; *p* < 0.001), social confinement (OR = 1.87, 95% CI, 1.33–2.62; *p* < 0.001), financial instability (OR = 2.38, 95% CI, 1.63–3.48; *p* < 0.001), and having non-specific psychological distress (K6 score ≥ 13) (OR = 4.84, 95% CI, 2.45–9.40; *p* < 0.001) (Additional file 2).

A hierarchical logistic regression analysis showed that stress in the work environment (OR = 2.09, 95% CI, 1.37–3.20; *p* < 0.001), financial instability (OR = 1.73, 95% CI, 1.12–2.67; *p* < 0.05), and low adherence to working remotely (OR = 1.65, 95% CI, 1.06–2.57; *p* < 0.05) were independently associated with poor sleep even after adjusting for the covariates (Table 1). Avoiding the three Cs was not significantly correlated with poor sleep after controlling for age and sex (Table 1). Nurses and other medically qualified professionals were younger than the other groups (median [interquartile range] age for physicians or dentists, 47.5 [36.0–56.3]; nurses, 40.0 [29.0–48.0]; other medically qualified professionals; 39.0 [32.0–45.0], and non-medically qualified professionals; 45.0 [39.0–53.0]). Most of the workers who could work remotely were non-medically qualified professionals, and none of the nurses could choose to work remotely (Additional file 3).

**Table 1:** Hierarchical logistic regression analysis of the incidence of poor sleep, COVID-19 preventive behaviors, and job-related stress in the

Category	Crude OR (95% CI)	*p*	Adjusted 1 OR (95% CI)	*p*	Adjusted 2 OR (95% CI)	*p*	Adjusted 3 OR (95% CI)	*p*
**COVID-19 preventive behavior (reference: high adherence)**								
Avoiding three Cs	0.70 (0.50–0.97)	< 0.05	0.70 (0.46–1.07)	n.s.	0.67 (0.44–1.03)	n.s.	0.70 (0.45–1.08)	n.s.
Maintaining a distance of at least one meter from others	0.93 (0.64–1.36)	n.s.	1.11 (0.70–1.77)	n.s.	1.19 (0.74–1.90)	n.s.	1.21 (0.75–1.94)	n.s.
Wearing a face mask regularly	1.03 (0.66–1.63)	n.s.	1.19 (0.70–2.03)	n.s.	1.26 (0.74–2.16)	n.s.	1.22 (0.71–2.11)	n.s.
Washing hands regularly	1.06 (0.68–1.65)	n.s.	1.06 (0.63–1.77)	n.s.	1.07 (0.63–1.79)	n.s.	1.02 (0.60–1.73)	n.s.
Working remotely	1.72 (1.17–2.53)	< 0.01	1.70 (1.11–2.60)	< 0.05	1.57 (1.02–2.43)	< 0.05	1.65 (1.06–2.57)	< 0.05
**Job-related stress under the COVID-19 pandemic (reference: less stressful)**								
Work environment	2.86 (2.01–4.06)	< 0.001	2.40 (1.59–3.62)	< 0.001	2.30 (1.52–3.49)	< 0.001	2.09 (1.37–3.20)	< 0.001
Exposure to patients	1.83 (1.22–2.73)	< 0.01	0.97 (0.59–1.58)	n.s.	0.92 (0.55–1.51)	n.s.	0.90 (0.54–1.51)	n.s.
Potential risk of COVID-19 infection	1.98 (1.41–2.78)	< 0.001	1.23 (0.76–1.99)	n.s.	1.23 (0.76–2.00)	n.s.	1.20 (0.74–1.96)	n.s.
Fear of infecting others	1.81 (1.29–2.54)	< 0.001	1.14 (0.71–1.83)	n.s.	1.13 (0.70–1.83)	n.s.	1.12 (0.69–1.82)	n.s.
Social confinement	1.87 (1.33–2.62)	< 0.001	1.29 (0.86–1.93)	n.s.	1.33 (0.88–2.01)	n.s.	1.32 (0.87–2.00)	n.s.
Financial instability	2.38 (1.63–3.48)	< 0.001	1.80 (1.18–2.74)	< 0.01	1.84 (1.20–2.83)	< 0.01	1.73 (1.12–2.67)	< 0.05

*Notes* Adjusted 1: Age and sex

Adjusted 2: Adjusted 1 + previously detected poor sleep-related covariates including existence of chronic diseases, being a frontline worker, and burden of caring for the elderly or children

Adjusted 3: Adjusted 2 + non-specific psychological distress accessed by using Kessler Psychological Distress Scale (K6)

*Abbreviations* OR, odds ratio; Three Cs, closed spaces, crowded places, and close contact; n.s., not significant

## Discussion

We conducted a cross-sectional examination of the prevalence of poor sleep and factors associated with poor sleep among HCWs in Japan in February 2021. The percentage of HCWs with poor sleep one year into the COVID-19 pandemic was still high (38.1%). In meta-analyses that include a number of studies using the PSQI, the crude estimated prevalence of poor sleep among healthcare professionals has been reported to be 47%: 31% after adjusting for publication bias [17]. The results of our study are relatively close to that estimation, suggesting that our results are consistent with previous studies. Among COVID-19 preventive behaviors, working remotely was protective against poor sleep. In line with this, stress in the work environment and financial instability exacerbated poor sleep.

### Job-related stresses during the COVID-19 pandemic

Stress in the work environment was independently associated with poor sleep even after controlling the covariates. Work environment stress, such as high work demands, job strain, bullying, and effort-reward imbalance, was reported to be related to poor sleep [29]. In our study, we did not ask about specific stressors in the workplace environment and therefore could not identify them. Our results show that stress in the work environment was important for HCWs during the COVID-19 pandemic. Thus, strategies that enhance the resilience of HCWs may be beneficial in preventing poor sleep [30]. 

The results also indicated a significant relationship between financial instability and poor sleep, consistent with previous population-based studies [31, 32]. Financial instability for participants and their families may include decreased income due to absence from work because of infection, increased health expenditures, and a rapid decline in the stock market. Several studies have implied the need for financial aid for HCWs [33]. Thus, a detailed examination of sleep and financial instability among HCWs is warranted.

### COVID-19 preventive behaviors

Among the five COVID-19 preventive behaviors, working remotely may have protected HCWs against poor sleep. During the COVID-19 pandemic, HCWs were severely afflicted with sleep problems compared to the general population [34], possibly owing to work-related stress, including exposure to patients with COVID-19 and conflict between work duties and family-related activities that the stay-at-home policy may have aggravated [35, 36]. Working remotely may facilitate protective effects against poor sleep by distancing these stresses. Furthermore, previous studies have emphasized the benefits of working remotely, including better mental health outcomes [37], ease of concentration on work and refreshment at home [37], and increased time spent with loved ones [38]. 

A study revealed that mental health professionals were satisfied with their full-time telemedicine experience [39], and the effort to have HCWs work remotely may contribute to health promotion. However, the background of participants who could choose to work remotely may differ from those who could not. For example, none of the nurses in this study could choose to work remotely. It should be recognized that some kinds of work cannot be done remotely. Thus, support from the work organization for preventing poor sleep quality among these professionals will be crucial, just as it is important to prevent burnout [40]. 

The relationship between poor sleep and other preventive measures, including high adherence to avoiding the three Cs, maintaining a distance of at least one meter from others, wearing a face mask, and washing hands regularly, was negligible after controlling for the covariates. This result is inconsistent with the results of a previous study that implicates a protective role against the severity of insomnia for washing hands [9]. This discrepancy may be due to differences in the participants’ profession and the survey’s timing. Unlike the previous study, which was conducted immediately after the pandemic and targeted the general workforce, this study was conducted with HCWs approximately a year after the pandemic began. HCWs were forced to adopt COVID-19 preventive behaviors in the early stages of the pandemic [41]. At the time of this study, however, such precautions may have become routine for the HCWs compared to at the beginning of the pandemic. Therefore, adherence to COVID-19 preventive behavior was not related to poor sleep at this stage. However, there may be various reasons behind preventive actions. Some optimistically believe in the preventive effects, while others may follow them due to anxiety and/or fear [42, 43]. Future studies to determine the association between preventive behaviors and poor sleep during a pandemic may benefit by examining the motivation for practicing preventive measures and the adherence rate.

### Limitation

This study has several limitations. First, it is cross-sectional, thus we cannot infer causal relationships. Second, sleep quality was measured using subjective measures. Considering the discrepancy in subjective-objective sleep [44], objective sleep measures, such as actigraphy and/or polysomnography, are needed to reinforce our results. Third, about half of the participants of this study were non-medical qualified staff who could have chosen to work remotely. Therefore, a careful comparison with other studies that included only physicians and nurses who cannot work remotely is needed. Fourth, the response rate for this survey was relatively low at 45.7%. This could potentially lead to non-response bias and therefore the results should be interpreted with caution. Fifth, alcohol consumption and smoking have been reported to be associated with poor sleep. While the rate of alcohol consumption among our study participants is comparable to that in the general population (12.1%),^22^ the rate of smoking among our study participants is relatively low compared to the general population (16.7%) but comparable to HCWs (8.2%) previously reported in Japan [45, 46]. In addition, sleep duration has been reported to be shorter in Japan than in other countries [47], and this trend could be worsening [48]. These could suggest that the participants of this study are at risk for chronic sleep deprivation, thus caution must be taken to applying the our results to HCWs in other countries.

## Conclusions

Unlike the previous literature, COVID-19 preventive behaviors, such as washing hands or avoiding the three Cs, were less associated with poor sleep in this study. Instead, stress due to the work environment had a greater association with the poor sleep of our HCWs. This study also suggests a possible protective effect against poor sleep for working remotely. Further research that examines methods to ameliorate the stress of HCWs who cannot work remotely is warranted.

### Electronic supplementary material

Below is the link to the electronic supplementary material.

Additional file: 1.docx. Participant characteristics.

Additional file: 2.docx. Univariate analysis of relative risk of poor sleep.

Additional file 3.docx. Type of work relation to poor-sleep related factors.

Additional file 4.docx. STROBE checklist.

## Data Availability

All data supporting this study’s findings are available from the corresponding author upon reasonable request with the approval of the ethics committee of the NCNP.

## Abbreviations

BMI: body mass index
CI: confidence interval
HCW: healthcare workers
K6: Kessler Psychological Distress Scale
NCNP: National Center of Neurology and Psychiatry
OR: odds ratio
PSQI: Pittsburgh Sleep Quality Index
Three Cs: closed spaces
Cp: crowded places
Cc: close contact
