# Supplementary material for: Association of COVID-19 preventive behavior and job-related stress with the sleep quality of healthcare workers one year into the COVID-19 outbreak: a Japanese cross-sectional survey
Source: Biopsychosoc Med. 2024 Mar 6;18:8. doi: 10.1186/s13030-024-00304-w (PMC10918958; doi:10.1186/s13030-024-00304-w)
Supplement: Supplementary file 1 — Additional file: 1.docx. Participant characteristics. [file 13030_2024_304_MOESM1_ESM.docx]

**Additional file 1. Participant characteristics**

| Category | Value | Total  N = 586 |
| --- | --- | --- |
| Sex | Female | 396 (67.6%) |
| Age | - | 43.0 (34.0–50.0) |
| BMI | - | 21.5 (19.9–23.7) |
| Smoking habit [cigarettes/day] | Regular smoker | 39 (6.7%) |
|  | Never | 430 (73.4%) |
|  | Quit | 108 (18.4%) |
|  | Occasionally | 9 (1.5%) |
|  | <10 | 16 (2.7%) |
|  | 11–20 | 22 (3.8%) |
|  | ≥21 | 1 (0.2%) |
| Alcohol consumption [g/day] | Habitual drinker | 190 (32.4%) |
|  | 10 g or less | 157 (26.8%) |
|  | 10–20 g | 133 (22.7%) |
|  | 20–40 g | 123 (21.0%) |
|  | 40–60 g | 42 (7.2%) |
|  | 60–80 g | 20 (3.4%) |
|  | ≥80 g | 5 (0.9%) |
| Exercise habit [minutes a week] | Regular exerciser | 98 (16.7%) |
|  | No | 116 (19.8%) |
|  | ≤30 | 171 (29.2%) |
|  | 31–59 | 94 (16.0%) |
|  | 60–119 | 107 (18.3%) |
|  | 120–179 | 40 (6.8%) |
|  | 180–239 | 26 (4.4%) |
|  | ≥240 | 32 (5.5%) |
| The number of people living with | 0 | 147 (25.1%) |
|  | 1 | 116 (19.8%) |
|  | 2 | 137 (23.4%) |
|  | 3 | 116 (19.8%) |
|  | 4 | 52 (8.9%) |
|  | ≥5 | 18 (3.1%) |
| Having the burden of caring for older adults or children | Yes | 85 (14.5%) |
| Type of profession | Physician or dentist | 42 (7.2%) |
|  | Nurse | 172 (29.4%) |
|  | Other medically qualified professionals | 116 (19.8%) |
|  | Non-medically qualified professionals | 256 (43.7%) |
| Average working hours per day | Long hour workers | 138 (23.5%) |
|  | <6 hours | 89 (15.2%) |
|  | 7 hours | 109 (18.6%) |
|  | 8 hours | 250 (42.7%) |
|  | 9 hours | 78 (13.3%) |
|  | 10 hours | 30 (5.1%) |
|  | 11 hours | 16 (2.7%) |
|  | 12 hours | 9 (1.5%) |
|  | ≥13 hours | 5 (0.9%) |
| Frontline worker | Yes | 66 (11.3%) |
| History of close contact with patients with COVID-19 | Yes | 19 (3.2%) |
|  | No | 513 (87.5%) |
|  | Not sure | 54 (9.2%) |
| Self-confinement due to possible infection of COVID-19 | Yes | 48 (8.2%) |
| Frequency of public transportation use [times a week] | Regular use | 252 (43.0%) |
|  | Less than once | 334 (57.0%) |
|  | Once or twice | 35 (6.0%) |
|  | Three or four times | 65 (11.1%) |
|  | Five or more | 152 (25.9%) |
| Experience of discrimination due to being a hospital worker | Yes | 38 (6.5%) |
| Existence of chronic disease | Yes | 169 (28.8%) |
| Hypertension | No | 524 (89.4%) |
|  | Yes (under medical treatment) | 42 (7.2%) |
|  | Yes (no treatment) | 15 (2.6%) |
| Diabetes mellitus | No | 570 (97.3%) |
|  | Yes (under medical treatment) | 7 (1.2%) |
|  | Yes (no treatment) | 2 (0.3%) |
| Respiratory disease | No | 542 (92.5%) |
|  | Yes (under medical treatment) | 14 (2.4%) |
|  | Yes (no treatment) | 23 (3.9%) |
| Heart failure | No | 570 (97.3%) |
|  | Yes (under medical treatment) | 6 (1.0%) |
|  | Yes (no treatment) | 4 (0.7%) |
| Cardiovascular disease | No | 579 (98.8%) |
|  | Yes (under medical treatment) | 1 (0.2%) |
| Cancer | No | 567 (96.8%) |
|  | Yes (under medical treatment) | 11 (1.9%) |
|  | Yes (no treatment) | 1 (0.2%) |
| Other | No | 498 (85.0%) |
|  | Yes (under medical treatment) | 64 (10.9%) |
|  | Yes (no treatment) | 10 (1.7%) |
| COVID-19 preventive behaviors | |  |
| Avoiding three Cs | Always | 301 (51.4%) |
|  | Often | 272 (46.4%) |
|  | Occasionally | 13 (2.2%) |
| Maintaining a distance of at least one meter from others | Always | 150 (25.6%) |
|  | Often | 377 (64.3%) |
|  | Occasionally | 57 (9.7%) |
|  | Never | 2 (0.3%) |
| Wearing a face mask regularly | Always | 493 (84.1%) |
|  | Often | 89 (15.2%) |
|  | Occasionally | 4 (0.7%) |
| Washing hands regularly | Always | 489 (83.4%) |
|  | Often | 92 (15.7%) |
|  | Occasionally | 5 (0.9%) |
| Working remotely | Always | 13 (2.2%) |
|  | Often | 53 (9.0%) |
|  | Occasionally | 98 (16.7%) |
|  | Never | 422 (72.0%) |
| Job-related stresses under the COVID-19 pandemic | |  |
| Work environment | Never | 60 (10.2%) |
|  | Slightly | 141 (24.1%) |
|  | Moderately | 176 (30.0%) |
|  | Severely | 161 (27.5%) |
|  | Very severely | 48 (8.2%) |
| Exposure to patients | Never | 233 (39.8%) |
|  | Slightly | 105 (17.9%) |
|  | Moderately | 125 (21.3%) |
|  | Severely | 95 (16.2%) |
|  | Very severely | 28 (4.8%) |
| Potential risk of COVID-19 infection | Never | 29 (4.9%) |
|  | Slightly | 108 (18.4%) |
|  | Moderately | 192 (32.8%) |
|  | Severely | 174 (29.7%) |
|  | Very severely | 83 (14.2%) |
| Fear of infecting others | Never | 30 (5.1%) |
|  | Slightly | 86 (14.7%) |
|  | Moderately | 174 (29.7%) |
|  | Severely | 191 (32.6%) |
|  | Very severely | 105 (17.9%) |
| Social confinement | Never | 35 (6.0%) |
|  | Slightly | 98 (16.7%) |
|  | Moderately | 186 (31.7%) |
|  | Severely | 187 (31.9%) |
|  | Very severely | 80 (13.7%) |
| Financial instability | Never | 130 (22.2%) |
|  | Slightly | 173 (29.5%) |
|  | Moderately | 136 (23.2%) |
|  | Severely | 98 (16.7%) |
|  | Very severely | 49 (8.4%) |
| Psychological distress (K6 score ≥ 13) | Distressed | 47 (8.0%) |
| Sleep disturbance | Poor sleep | 223 (38.1%) |

**Abbreviations:** BMI, body mass index; Three Cs, closed spaces, crowded places, and close contact
