# Supplementary material for: Association of COVID-19 preventive behavior and job-related stress with the sleep quality of healthcare workers one year into the COVID-19 outbreak: a Japanese cross-sectional survey
Source: Biopsychosoc Med. 2024 Mar 6;18:8. doi: 10.1186/s13030-024-00304-w (PMC10918958; doi:10.1186/s13030-024-00304-w)
Supplement: Supplementary file 2 — Additional file: 2.docx. Univariate analysis of relative risk of poor sleep. [file 13030_2024_304_MOESM2_ESM.docx]

**Additional file 2. Univariate analysis of relative risk of poor sleep**

| Category | Univariate analysis OR (95 % CI) | *p* |
| --- | --- | --- |
| Sex (reference: female) | 1.09 (0.77–1.56) | n.s. |
| Age | 1.02 (1.01–1.04) | < 0.01 |
| BMI | 1.06 (1.01–1.11) | < 0.05 |
| Smoking habits | 1.60 (0.83–3.06) | n.s. |
| Alcohol consumption | 1.25 (0.87–1.77) | n.s. |
| Exercise habits | 0.98 (0.63–1.54) | n.s. |
| Number of people living with | 0.98 (0.87–1.10) | n.s. |
| Having the burden of caring for older adults or children | 0.82 (0.51–1.33) | n.s. |
| Type of profession (reference: doctor) |  | n.s. |
| Nurse | 1.29 (0.64–2.57) | n.s. |
| Other medical staff | 0.96 (0.46–1.98) | n.s. |
| Non-medical staff | 0.84 (0.43–1.65) | n.s. |
| Work hours (reference: 8 hours or less) | 1.24 (0.84–1.83) | n.s. |
| Being a frontline worker | 1.73 (1.03–2.89) | < 0.05 |
| History of close contact with COVID-19 patients (reference: no) | | |
| Yes | 1.94 (0.77–4.85) | n.s. |
| Not sure | 1.62 (0.92–2.84) | n.s. |
| Self-confinement due to possible infection of COVID-19 | 1.55 (0.86–2.81) | n.s. |
| Regular use of public transportation | 0.86 (0.62–1.21) | n.s. |
| Existence of chronic diseases | 1.66 (1.16–2.39) | < 0.01 |
| Experience of discrimination due to being a hospital worker | 2.10 (1.08–4.07) | < 0.05 |
| COVID-19 preventive behaviors (reference: high adherence) | | |
| Avoiding three Cs | 0.70 (0.50–0.97) | < 0.05 |
| Maintaining a distance of at least one meter from others | 0.93 (0.64–1.36) | n.s. |
| Wearing a face mask regularly | 1.03 (0.66–1.63) | n.s. |
| Washing hands regularly | 1.06 (0.68–1.65) | n.s. |
| Working remotely | 1.72 (1.17–2.53) | < 0.01 |
| Job-related stresses under the COVID-19 pandemic (reference: less stressful) | | |
| Work environment | 2.86 (2.01–4.06) | < 0.001 |
| Exposure to patients | 1.83 (1.22–2.73) | < 0.01 |
| Potential risk of COVID-19 infection | 1.98 (1.41–2.78) | < 0.001 |
| Fear of infecting others | 1.81 (1.29–2.54) | < 0.001 |
| Social confinement | 1.87 (1.33–2.62) | < 0.001 |
| Financial instability | 2.38 (1.63–3.48) | < 0.001 |
| Psychological distress (K6 score ≥ 13) | 4.84 (2.45–9.40) | < 0.001 |

**Abbreviations:** OR, odds ratio; BMI, body mass index; Three Cs, closed spaces, crowded places, and close contact; n.s., not significant
