# Supplementary material for: Association of COVID-19 preventive behavior and job-related stress with the sleep quality of healthcare workers one year into the COVID-19 outbreak: a Japanese cross-sectional survey
Source: Biopsychosoc Med. 2024 Mar 6;18:8. doi: 10.1186/s13030-024-00304-w (PMC10918958; doi:10.1186/s13030-024-00304-w)
Supplement: Supplementary file 3 — Additional file 3.docx. Type of work relation to poor-sleep related factors. [file 13030_2024_304_MOESM3_ESM.docx]

**Additional file 3. Relation of type of work to poor-sleep related factors**

| Types of work | Physician or dentist  n = 42 | Nurse  n = 172 | Other medically qualified professionals  n = 116 | Non-medically qualified professionals  n = 256 |
| --- | --- | --- | --- | --- |
| Age [median (interquartile range)] | 47.5 (36.0–56.3) | 40.0 (29.0–48.0) | 39.0 (32.0–45.0) | 45.0 (39.0–53.0) |
| BMI [median (interquartile range)] | 22.9 (20.9–24.8) | 21.5 (19.9–23.7) | 21.2 (19.5–23.0) | 21.5 (19.8–23.8) |
| Having the burden of caring for older adults or children | 7 (16.7%) | 18 (10.5%) | 24 (20.7%) | 36 (14.1%) |
| Being a frontline worker | 10 (23.8%) | 43 (25.3%) | 11 (9.6%) | 2 (0.8%) |
| Existence of chronic diseases | 16 (38.1%) | 51 (29.7%) | 30 (25.9%) | 72 (28.1%) |
| Experience of discrimination due to being a hospital worker | 3 (7.3%) | 20 (11.6%) | 5 (4.3%) | 10 (3.9%) |
| COVID-19 preventive behavior | | | | |
| Avoiding three Cs | 28 (66.7%) | 73 (42.4%) | 55 (47.4%) | 145(56.6%) |
| Maintaining a distance of at least one meter from others | 20 (47.6%) | 32 (18.6%) | 26 (22.4%) | 72(28.1%) |
| Wearing a face mask regularly | 36 (85.7%) | 141 (82.0%) | 91 (78.4%) | 225(87.9%) |
| Washing hands regularly | 34 (81.0%) | 140 (81.4%) | 97 (83.6%) | 218(85.2%) |
| Working remotely | 7 (16.7%) | 0 (0.0%) | 23 (20.0%) | 134(52.3%) |
| Job-related stress under the COVID-19 pandemic | | | | |
| Work environment | 10 (23.8%) | 89 (51.7%) | 45 (38.8%) | 65(25.4%) |
| Exposure to patients | 6 (14.3%) | 86 (50.0%) | 20 (17.2%) | 11(4.3%) |
| Potential risk of COVID-19 infection | 6 (14.3%) | 94 (54.7%) | 48 (41.4%) | 109(42.6%) |
| Fear of infecting others | 14 (33.3%) | 104 (60.5%) | 65 (56.0%) | 113(44.1%) |
| Social confinement | 11 (26.2%) | 97 (56.4%) | 56 (48.3%) | 103(40.2%) |
| Financial instability | 1 (2.4%) | 49 (28.5%) | 27 (23.3%) | 70(27.3%) |
| Psychological distress (K6 score ≥ 13) | 1 (2.4%) | 18 (10.5%) | 10 (8.6%) | 18 (7.1%) |
| PSQI global score [mean (standard deviation)] | 5.0 (3.1) | 5.7 (3.3) | 4.9 (2.3) | 4.8 (2.8) |

**Abbreviations**: BMI: body mass index; Three Cs: closed spaces, crowded places, and close contact
