# Supplementary material for: Association of COVID-19 preventive behavior and job-related stress with the sleep quality of healthcare workers one year into the COVID-19 outbreak: a Japanese cross-sectional survey
Source: Biopsychosoc Med. 2024 Mar 6;18:8. doi: 10.1186/s13030-024-00304-w (PMC10918958; doi:10.1186/s13030-024-00304-w)
Supplement: Supplementary file 4 — Additional file 4.docx. STROBE checklist. [file 13030_2024_304_MOESM4_ESM.docx]

**Additional file 4.**

**STROBE Statement: checklist of items that should be included in reports of observational studies**

|  | Item No | Recommendation | Authors’ comment |
| --- | --- | --- | --- |
| **Title and abstract** | 1 | (*a*) Indicate the study’s design with a commonly used term in the title or the abstract | The title includes “a cross-sectional survey in Japan.” |
|  |  | (*b*) Provide in the abstract an informative and balanced summary of what was done and what was found | An informative and balanced summary is provided. |
| Introduction | | |  |
| Background/rationale | 2 | Explain the scientific background and rationale for the investigation being reported | The scientific background and rationale for the investigation have been described in the Introduction section (page 3). |
| Objectives | 3 | State specific objectives, including any prespecified hypotheses | Objectives and hypotheses have been described at the end of the Introduction section (page 4). |
| Methods | | |  |
| Study design | 4 | Present key elements of study design early in the paper | Study design has been described at the beginning of the Methods section (pages 4–7). |
| Setting | 5 | Describe the setting, locations, and relevant dates, including periods of recruitment, exposure, follow-up, and data collection | Study setting, locations, and relevant dates have been described at the beginning of the Methods section (pages 4–7). |
| Participants | 6 | (*a*) *Cohort study*—Give the eligibility criteria, and the sources and methods of selection of participants. Describe methods of follow-up  *Case-control study*—Give the eligibility criteria and the sources and methods of case ascertainment and control selection. Give the rationale for the choice of cases and controls  *Cross-sectional study*—Give the eligibility criteria, and the sources and methods of selection of participants | All workers of the National Center Hospital, National Center of Neurology and Psychiatry (NCNP) were invited, as shown in Figure 1. |
|  |  | (*b*) *Cohort study*—For matched studies, give matching criteria and number of exposed and unexposed  *Case-control study*—For matched studies, give matching criteria and the number of controls per case | Not relevant |
| Variables | 7 | Clearly define all outcomes, exposures, predictors, potential confounders, and effect modifiers. Give diagnostic criteria, if applicable | All outcomes have been described in the Methods section (pages 4–7). |
| Data sources/ measurement | 8* | For each variable of interest, give sources of data and details of methods of assessment (measurement). Describe comparability of assessment methods if there is more than one group | Sources of data and details of methods of assessment have been described in the Methods section (pages 4–7). |
| Bias | 9 | Describe any efforts to address potential sources of bias | Potential sources of bias have been discussed in the Limitation section (page 12). |
| Study size | 10 | Explain how the study size was arrived at | Sample size calculation has been described in the Statistics section (page 7). |
| Quantitative variables | 11 | Explain how quantitative variables were handled in the analyses. If applicable, describe which groupings were chosen and why | The handling of quantitative variables has been described in the Methods section (pages 4–7). |
| Statistical methods | 12 | (*a*) Describe all statistical methods, including those used to control for confounding | All statistical methods have been described in the Statistics section (page 7). |
|  |  | (*b*) Describe any methods used to examine subgroups and interactions | Not relevant. |
|  |  | (*c*) Explain how missing data were addressed | A complete case analysis was conducted, as described in the Statistics section (page 7). |
|  |  | (*d*) *Cohort study*—If applicable, explain how loss to follow-up was addressed  *Case-control study*—If applicable, explain how matching of cases and controls was addressed  *Cross-sectional study*—If applicable, describe analytical methods taking account of sampling strategy | Not relevant. |
|  |  | (*e*) Describe any sensitivity analyses | Not relevant. |
| **Results** |  |  |  |
| Participants | 13* | (a) Report numbers of individuals at each stage of study—eg numbers potentially eligible, examined for eligibility, confirmed eligible, included in the study, completing follow-up, and analysed | The number of inclusions has been described in Figure 1. |
|  |  | (b) Give reasons for non-participation at each stage | Not relevant. |
|  |  | (c) Consider use of a flow diagram | Figure 1 is a flow diagram. |
| Descriptive data | 14* | (a) Give characteristics of study participants (eg demographic, clinical, social) and information on exposures and potential confounders | Characteristics of the study participants are shown in Additional file 1. |
|  |  | (b) Indicate number of participants with missing data for each variable of interest | The number of participants with missing data has been described in Figure 1. |
|  |  | (c) *Cohort study*—Summarise follow-up time (eg, average and total amount) | Not relevant. |
| Outcome data | 15* | *Cohort study*—Report numbers of outcome events or summary measures over time | Not relevant. |
|  |  | *Case-control study—*Report numbers in each exposure category, or summary measures of exposure | Not relevant. |
|  |  | *Cross-sectional study—*Report numbers of outcome events or summary measures | The number of participants with poor sleep is reported in the Results section (pages 8–10). |
| Main results | 16 | (*a*) Give unadjusted estimates and, if applicable, confounder-adjusted estimates and their precision (eg, 95% confidence interval). Make clear which confounders were adjusted for and why they were included | Unadjusted estimates and confounder-adjusted estimates with a 95% confidence interval are given in Table 1. |
|  |  | (*b*) Report category boundaries when continuous variables were categorized | Category boundaries are reported in the Methods section (pages 4–7). |
|  |  | (*c*) If relevant, consider translating estimates of relative risk into absolute risk for a meaningful time period | Not relevant. |
| Other analyses | 17 | Report other analyses done—eg analyses of subgroups and interactions, and sensitivity analyses | Not relevant. |
| Discussion |  |  |  |
| Key results | 18 | Summarise key results with reference to study objectives | Key results have been summarized at the beginning of the Discussion section (page 10). |
| Limitations | 19 | Discuss limitations of the study, taking into account sources of potential bias or imprecision. Discuss both direction and magnitude of any potential bias | Limitations have been discussed in the Limitation section (page 12). |
| Interpretation | 20 | Give a cautious overall interpretation of results considering objectives, limitations, multiplicity of analyses, results from similar studies, and other relevant evidence | A cautious interpretation has been made in the Discussion section (pages 10–13). |
| Generalisability | 21 | Discuss the generalisability (external validity) of the study results | The generalisability is discussed in the Discussion section (pages 10–13). |
| Other information |  |  |  |
| Funding | 22 | Give the source of funding and the role of the funders for the present study and, if applicable, for the original study on which the present article is based | The source of funding and the role of the funders is described in the Declarations section (page 14). |

*Give information separately for cases and controls in case-control studies and, if applicable, for exposed and unexposed groups in cohort and cross-sectional studies.

**Note:** An Explanation and Elaboration article discusses each checklist item and gives methodological background and published examples of transparent reporting. The STROBE checklist is best used in conjunction with this article (freely available on the Web sites of PLoS Medicine at http://www.plosmedicine.org/, Annals of Internal Medicine at http://www.annals.org/, and Epidemiology at http://www.epidem.com/). Information on the STROBE Initiative is available at www.strobe-statement.org.
